# Living a private lie: intersectional stigma, depression and suicidal thoughts for selected young key populations living with HIV in Zambia

**DOI:** 10.1186/s12889-024-19278-z

**Published:** 2024-07-19

**Authors:** Joseph Mumba Zulu, Henna Budhwani, Bo Wang, Anitha Menon, Deogwoon Kim, Mirriam Zulu, Patrick Nyamaruze, Kaymarlin Govender, Russell Armstrong

**Affiliations:** 1 Department of Health Policy and Management, School of Public Health, University of Zambia, PO Box 50110, Lusaka, Zambia; 2 Center for Community Health Systems and Implementation Research, Lusaka, Zambia; 3 Institute on Digital Health and Innovation, College of Nursing, Florida State University, Tallahassee, FL USA; 4 Department of Population and Quantitative Health Sciences, UMass Chan Medical, School 368 Plantation Street, Worcester, MA 01605 US; 5 Department of Psychology, School of Humanities and Social Sciences, University of Zambia, Box 32379, Lusaka, Zambia; 6 Psychology Program, School of Liberal Studies, University of Petroleum and Energy Sciences, Dehradun, India; 7 Liberal Arts Department, Rochester Institute of Technology, Dubai, UAE; 8 Health Economics and HIV/AIDS Research Division, University of KwaZulu-Natal, Durban, South Africa

**Keywords:** Intersectional stigma, Depression, Suicidal thoughts, Young key populations living with HIV, Zambia

## Abstract

**Background:**

Limited research has been conducted on the forms, manifestations and effects of intersectional stigma among young HIV-positive men who have sex with men (MSM) and transgender women (TGW) in Zambia. In this study, we aimed to address this gap by elucidating the experiences of these in a small group of young, HIV + MSM and TGW in Zambia.

**Methods:**

We applied a mixed-methods design. Data were collected from January 2022 to May 2022. Qualitative data were collected using in-depth interviews while quantitative data were collected using a questionnaire. Qualitative transcripts were coded using thematic analysis while paper-based questionnaire data were entered into Kobo Connect. Descriptive statistics, using chi-squared tests were calculated using Excel. In this paper, we provide a descriptive profile of the sample and then focus on the qualitative findings on intersectional stigma, depression, and contemplation of suicide.

**Results:**

We recruited 56 participants from three sites: Lusaka, Chipata, and Solwezi districts. Participants’ mean age was 23 years. The study found that 36% of all participants had moderate to significant symptoms of depression, 7% had major depression, 30% had moderate signs of anxiety, 11% had high signs of anxiety, 4% had very high signs of anxiety and 36% had contemplated suicide at least once. A greater proportion of TGW had moderate to significant symptoms of depression (40%) or major depression (10%) compared to MSM, at 33% and 6%, respectively (*X*^2^ = 0.65; *p* = 0.42). Similarly, more TGW (55%) had contemplated suicide than MSM peers (36%, *X*^2^=1.87; *p* = 0.17). In the qualitative data, four emergent themes about the forms, manifestations, and effects of intersectional stigma were (1) HIV, sexual orientation, and gender identity disclosure; (2) Dual identity; (3) Challenges of finding and maintaining sexual partners; (4) Coping and resilience. Overall, having to hide both one’s sexuality and HIV status had a compounding effect and was described as living *“a private lie.”*

**Conclusion:**

Effectively addressing stigmas and poor mental health outcomes among young HIV-positive MSM and TGW will require adopting a socio-ecological approach that focuses on structural interventions, more trauma-informed and identity-supportive care for young people with HIV, as well as strengthening of authentic community-informed public health efforts.

## Introduction

Sub-Saharan Africa (SSA) is severely affected by the HIV epidemic, with Zambia being one of the most affected countries [1, 2]. The most current (2018) Zambia Demographic Health Survey indicates that the adult (15–49 years) HIV prevalence was 11.1% in Zambia [3]. The higher rates were among key populations reaching 21% among men who have sex with men (MSM) and 22% among transgender women (TGW) in 2020 [4]. While coverage of life-saving antiretroviral therapy (ART) was estimated to be 90% of all people living with HIV (PLHIV) in SSA, significant inequities remain in enrolling and sustaining people on ART [1, 2]. There are significant variations in the estimates across countries [1, 2]. Even though key populations accounted for 46% of new HIV infections across southern Africa in 2022 [5], MSM and TGW tend to be the least targeted by programs [6]. There are disparities in programs targeting and supporting alleviation of HIV-related health burden among MSM and TGW who simultaneously face various challenges, including mental health issues and stigmas, compounding their overall health vulnerabilities [1, 2, 7].

Young MSM and TGW, compared to the young heterosexual group, experience more mental health challenges [8–10], arising from victimization, bullying, internalized homophobia and discrimination [11], violence, socio-cultural and religious attitudes regarding sexual or gender diversity [12], and criminalization of sexual or gender diversity in Zambia and other African settings [10, 13]. Sections 155–157 of the Zambian Penal Code criminalize any form of consensual same- sex conduct [10, 13]. Legal challenges and socio-cultural contextual barriers such as high risks of stigma, discrimination, and violence linked to the criminalization of practices regarding sexual or gender diversity tend to compound mental health challenges within this population [14]. These mental health stressors negatively interact with important HIV-related health outcomes, including uptake and retention in HIV programmes, medication adherence, and achieving and sustaining viral suppression [15, 16]. It is important to understand in detail the mental health issues in this group given that the developmental stage is associated with increased independence, risk-taking, and changing social support [17].

Young key populations living with HIV (YPLWH) aged between 18 and 24 years face disproportionate HIV stigma [18, 19] that negatively affects HIV health management [19]. In addition to HIV stigma, some of these key populations have to also deal with sexual orientation and gender identity (SOGI) related stigma [19]. The convergence of multiple stigmatized identities within a person or group, or intersectional stigma tends to further worsen health outcomes in key populations [20]. An intersectional perspective provides an opportunity to think holistically about how living with multiple stigmatized identities affects behaviours, and different health outcomes [21], and to critically examine how systems of oppression interact at the societal, community, and individual levels [22].

Country -specific knowledge about the forms, manifestations and effects of intersectional stigma as experienced by young MSM and TGW with HIV in Zambia is beginning to emerge, largely relying on survey-based, cross-sectional research [3]. How contextual factors work as drivers of stigma, and how these drivers then manifest themselves as intersectional stigma in the lives of young MSM and TGW with HIV and the consequences of this manifestation including negative influences on different aspects of mental health has not been fully explored especially for HIV-positive MSM [23], and TGW in African settings [24, 25]. It is critical to consider multiple stigmatized identities in order to develop effective intervention strategies and improve the psychosocial well-being of marginalized populations [26].

While there is a substantive presence of sexual and gender minorities in Zambia, very little research in any form has emerged [10]. As the country increases its efforts to address HIV and to improve its capacity to reach or exceed its 2030 targets and commitments, it becomes important to address these knowledge gaps. The target is to test 95% of all PLHIV, have 95% of those diagnosed on ART and achieve viral suppression for 95% by 2025 in order to end AIDS by 2030 [10]. This study used a mixed-methods design to arrive at an intersectional view of the experiences of young, HIV-positive MSM and TGW in Zambia to understand how intersectional stigma is constructed and how it affects mental health outcomes in Zambia. This study is part of the regional study on exploring the influence of intersectional stigma on uptake and retention in ART programmes for selected key population groups in three Southern African Development Community (SADC) countries – Malawi, Zambia and Zimbabwe.

The conceptual framework for the research draws on emerging trends in stigma theory and research, including the concepts of minority stress, intersectionality and intersectional stigma [27, 28]. In the framework, stigma is defined as the “the co-occurrence of labelling, stereotyping, separation, status loss, and discrimination in a context in which power is exercised” [29]. In this view, stigma is a product of social and structural relations where status and value are contested and resolved through gains and losses in social position and worth. This is similar to Parker and Aggleton who describe HIV-related stigma and discrimination as “social processes linked to the reproduction of inequality and exclusion” [28]. While the mechanics of stigma may emanate from structural forces, what matters most is how these interact with and affect the health and well-being of individuals in communities [28].

### Intersectional stigma, sexual and gender diversity and HIV: a conceptual framework

**Fig. 1:**
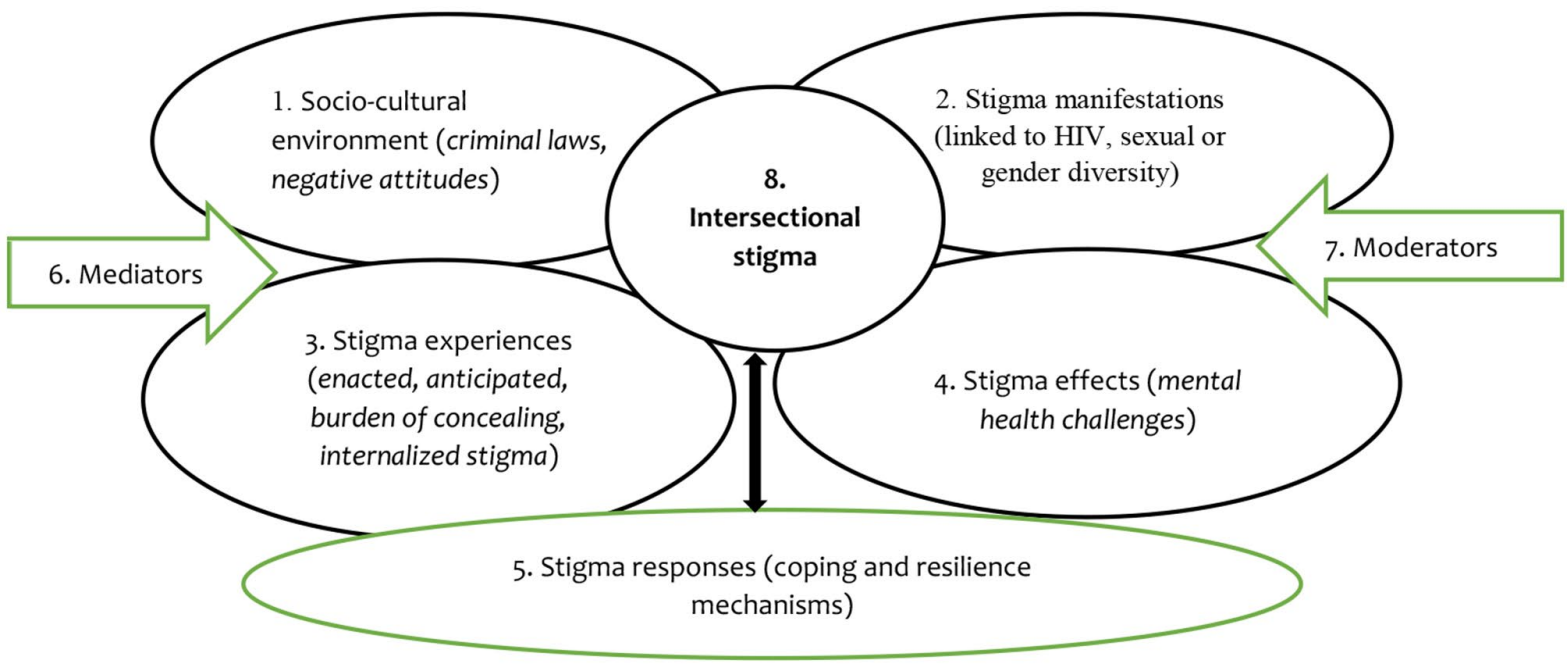
Conceptual framework

Given this range and depth of issues needing further exploration, a flexible conceptual framework was proposed for this research project. While, on the one hand, it took account of evolving theory and practice related to stigma in the southern African region and beyond, it did not pre-define or preclude the emergence of a more nuanced and context-specific understanding given the dearth of previous research to date that asks similar questions( Fig. 1, below):

At the outset, the social environment (1) in Zambia included social and structural processes that may drive and perpetuate stigma or prevent or limit stigma. Within this context, stigma manifests itself (2) and is experienced by individuals (3) in a variety of forms, largely as either enacted stigma, anticipated or feared stigma, or internalised stigma. The effects of stigma is felt by individuals (4) with a range of physical, mental or social consequences. The individuals respond with different strategies and behaviours to cope with these effects (5), to become resilient, or to resist and transform stigma [13, 30]. One measure of the success of these efforts is behavioural intentions to enrol in and/or remain in HIV care and intentions towards positive prevention [22]. How these links and pathways operate for individuals are influenced by mediators and moderators. Mediators (6) intensity stigma effects [13]. Moderators (7) operate more broadly across the framework and include an individual’s background conditions or starting point as experiences of stigma accumulate [13]. An intersectional approach (8) posits that all of the relationships and linkages described previously are “interdependent and mutually constitutive” and that these generate some degree of “causal complexity” [31]. Intersectionality opens up the possibility that such interactions are, “synergistic, producing different and distinct experiences of oppression and opportunity“ [32].

## Methodology

### Study design

The study followed a mixed-methods, parallel design with simultaneous collection of qualitative and quantitative data from the same study sample. Mixed methods designs are preferable for intersectionality research since intersectionality is conceptualised as a multilevel and multidimensional framework [33, 34]. The different methodologies may be advantageous as they might effectively apply to the different levels and particular features of intersectionality [33, 34]. Using the two approaches allowed for triangulation and a richer analysis of the complex phenomena underlying intersectional stigma for the study participants [30, 35]. This study largely reports qualitative data on experiences of intersectional stigma as well as depression and suicide thoughts. The qualitative study component adopted a phenomenological study design as the aim was to document experiences of mental health and causes of mental health among young, HIV positive MSM and TGW [36].

### Study population

The study population involved young MSM and TGW in Zambia, all self-disclosed as HIV-positive and currently on ART. The inclusion criteria consisted of age (18 to 24 years); self-identifying as gay, bisexual or MSM, or TGW or female with a gender at birth being male; self-identifying as HIV-positive; and self-identifying as currently on ART. All potential participants who were screened for eligibility enrolled or agreed to participate in the study. Participants who did not meet all of these criteria, as administered through an eligibility test at the start of the data collection encounter, were excluded. At total of 56 young people agreed to participate and completed both the questionnaire and the in-depth interview. There were 29 from Lusaka, 14 from Chipata and 13 from Solwezi. Lusaka was selected because it is the capital city of Zambia. Chipata was included as it is a border town while Solwezi is an emerging mining town. Further, these districts are among those with the highest HIV cases.

Given the Zambian context where sexual/ gender diversity has been criminalized, and the lack of previous research experience within the study population, snow-ball sampling was used to recruit potential participants in three locations (Chipata, Lusaka and Solwezi) [10]. This is a sampling method in which one interviewee provides a name of at least one more potential interviewee; and in turn this interviewee also recommends another or more potential interviewees, and so on [37]. Before recruiting participants, a mobiliser (a member of the MSM community) and members of the peer interview team confidentially promoted the study within their different social networks. In addition, organisations providing HIV services were also contacted to promote the study and encourage participation. To reduce bias, we started with a sample with seeds that were as diverse as possible. This was done by recruiting participants through seven peer interview team members [37].

### Data collection

Data collection occurred in secure, confidential settings. Data were collected from January 2022 to May 2022. Locations were chosen based on the recommendations of the study team and by representatives of sexual minority organisations consulted during the study design. The qualitative component involved semi-structured interviews while the quantitative component involved a self-administered, confidential questionnaire. The data collection tools were translated into the local languages (Nyanja and Bemba). The interviews were conducted once. Each participant was screened in terms of eligibility criteria and then was asked to complete informed consent form. A survey was administered followed by short break and then a semi-structured interview. Each survey took about 30 min while the semi-structured interviews ranged between 35 min and 1 h. We provided for a 30 min break between the survey and semi-structured interviews to ensure that the timing of the survey and semi-structured interviews did not compromise participant response. During the break, the respondent was provided with refreshments.

The self-administered survey was constructed of multiple-choice, closed-ended items that gathered data on SOGI, socio-economic characteristics, living arrangements and relationships status, length of time living with HIV, preferred providers for HIV services, self-reported ART adherence and knowledge of viral suppression. Participants were asked ‘Do you consider yourself as: male, female, which we said was ‘self-defined’. In terms of sexual orientation, participants had five choices: gay, bisexual, transgender, heterosexual, and other. No TGW identified as heterosexual. It also included sections on alcohol and drug use (using the Alcohol Use Disorders Identification Test [AUDIT] and the Drug Use Disorders Identification Test [DUDIT]), and experiences with physical or sexual violence (having experienced violence or having done such things to others) [38, 39]. Three specific items addressed mental health: the Center for Epidemiological Studies-Depression (CES-D)-10 scale, the General Anxiety Disorder (GAD)-7 scale, and the Suicide Behaviors Questionnaire-Revised (SBQ-R), all of which had been previously validated in Zambia or a similar African setting although not with the specific population for this study [40–42]. Additional sections addressed experiences of stigma and discrimination related to SOGI and HIV status using questions adapted from the PLHIV Stigma Index 2.0 tool [23].

To provide insight into the potential effects of intersectional stigma on an individual’s mental well-being, as part of the questionnaire, participants completed the CESD-10 (symptoms of depression) and the SBQ-R screening tools (thoughts and experiences of suicide) [40–42]. According to the CESD-10 methodology, a score of 0–10 means low to no symptoms of depression, 11–15 moderate symptoms of depression, and 16–25 moderate to severe symptoms of depression [40–42].

The semi structured guide explored the following topics: experiences growing up in family and community; future expectations; self-concept, including sexuality, gender identity and HIV status; strategies for day-to-day living, including managing disclosure, protection of social status and resilience; negative experiences (stigma, discrimination or violence, actual or feared) related to SOGI and HIV status; effects of stigma and discrimination on mental health, and coping and recovering strategies, including experiences seeking mental health support; experiences with ART, including adherence enablers and challenges; and, finally, ideas for change and improvement. The interviews were conducted by experienced members of the key populations network who had been engaged as research assistants in two other studies on young key populations. The research assistants also participated in transcribing the data and data validation process.

### Data analysis

For qualitative data, audio recordings were transcribed verbatim by the trained research team. Thematic analysis based on structural coding aligned to the conceptual framework was used to analyse the data [43]. Data saturation, which is the stage when no additional new information can be attained was reached during data collection [44]. This was discussed with the data collection team and also validated during coding process in the coding team. The two lead investigators developed a coding manual and initially coded four transcripts (JMZ and RA). The manual and coded transcripts were reviewed by a third researcher for clarity and consistency (PN). Subsequently, the code book and all transcripts were loaded to Nvivo (12 pro). Transcripts were independently coded with periodic quality assurance checks using Nvivo.

For quantitative data, survey results were entered into Excel and analysed using descriptive statistical techniques to generate descriptors and other insights regarding the study participants on key dimensions linked to the conceptual framework. The paper survey results were entered into Kobo Connect by a data manager. Periodic quality assurance checks were performed by the research coordinator to minimize data entry errors. Chi-square tests (including fisher’ exact tests) to examine the differences in proportions between the two groups were done. Excel data were converted into SAS data for analysis. Statistical significance was set at *p* < 0.05. A validation workshop was held in Lusaka in order to validate the findings of the study.

### Ethics

This study was approved by the Research Ethics Committee of the University of Zambia, the National Health Research Authority as well as Biomedical Health Research Ethics Committee of the University of KwaZulu-Natal. At no time was personal identifying information collected. Following administration of an eligibility assessment and verbal informed consent procedures, participants completed an anonymous questionnaire in English and placed it in a sealed envelope. Subsequently, participants were asked again to consent to audio recording and, if agreeable, proceeded through a semi-structured, in-depth interview lasting between 30 and 40 min. Participants in the study volunteered to take part, and interviewees had the freedom to withdraw from the study at any time. Additionally, participants were given details about counselling and care referral options if needed.

## Results

The results section has been divided into quantitative and qualitative research sections. The quantitative results are presented first.

### Quantitative results

The characteristics of the study’s participants are summarised in Table 1 below.

**Table 1:** Characteristic of participants

Characteristic	Total *N*(%)	MSM *N*(%)	TG *N*(%)	*P*
**N=**	56	36	20	
**Age (Med)**	23	22	23	
Missing	2	0	2	
**Gender (at birth)**				
Male	56	36	20	
**Gender (self-defined)**				
Male	38 (68%)	36 (100%)	2 (10%)	< 0.001
Female	7 (12%)	0	7 (35%)	
Transgender	11 (20%)	0	11 (55%)	
**Sexual orientation**				
Gay/homosexual	32 (57%)	28 (78%)	4 (20%)	< 0.001
Bisexual	8 (14%)	8 (22%)	0	
Transgender	16 (29%)	0	16 (80%)	
**Education**				
Primary	9 (16%)	6 (17%)	3 (15%)	0.654
Secondary	29 (52%)	17 (47%)	12 (60%)	
Post-secondary	18 (32%)	13 (36%)	5 (25%)	
**Employment**				
Employed (FT/PT)	13 (23%)	6 (17%)	7 (35%)	0.161
Employed (Self)	11 (20%)	6 (17%)	5 (25%)	
Not employed	24 (43%)	17 (47%)	7 (35%)	
Student	8 (14%)	7 (19%)	1 (5%)	
**Social status**				
Single	29 (52%)	18 (50%)	11 (55%)	0.720
Relationship	27 (48%)	18 (50%)	9 (45%)	
**Housing (living with)**				
Family	26 (46%)	18 (50%)	8 (40%)	0.116
Alone	11 (21%)	4 (11%)	7 (35%)	
With friends	15 (27%)	10 (28%)	5 (25%)	
Student	1 (1%)	3 (8%)	0	
Homeless	1 (1%)	1 (3%)	0	
**Religion**				
Christian	53 (92%)	33 (92%)	20 (100%)	0.697
Muslim	1 (2%)	1 (2%)	0	
None	2 (6%)	2 (6%)	0	

Of the total number of participants, 64% (36) initially identified as MSM and 36% (20) as transgender women. In terms of self-assigned gender, two thirds (66%) defined themselves as male, 12% as female, and 22% as transgender. With regard to sexual orientation, over half of the participants (55%) described themselves as gay; eight participants (14%) described themselves as bisexual. The remaining 31% identified themselves as transgender women with primarily male sexual partners (Table 1).

More than two-thirds of participants (69%) indicated that they had been diagnosed with HIV within the past two years. One TGW participant indicated that she had acquired HIV at birth. Almost three-quarters (72%) indicated they had been on ART for two years or less. Almost half of participants (47%) were diagnosed at government facilities and a similar proportion (50%) indicated that they were also receiving their ongoing care at these facilities. Place of service was mainly government facilities or NGO facilities-with higher proportion of MSM visiting these services (Table 2).

**Table 2:** HIV-related characteristics of participants

HIV diagnosis	Total *N*(%)	MSM *N*(%)	TG *N*(%)	Chi-squared *p* value	ART	Total *N*(%)	MSM *N*(%)	TG *N*(%)	Chi-squared *p* value
**N=**	56	36	20		N=	56	36	20	
**Time since diagnosis (years)**					**Time on ART (years)**				
5+	8 (15%)	6 (17%)	2 (10%)	0.728	5+	8 (14%)	6 (17%)	2 (10%)	0.810
4	3 (5%)	1 (3%)	2 (10%)		4	3 (5%)	1 (3%)	2 (10%)	
3	5 (11%)	4 (11%)	1 (5%)		3	5 (9%)	4 (11%)	1 (5%)	
2	16 (25%)	9 (25%)	7 (35%)		2	16 (29%)	9 (25%)	7 (35%)	
1	12 (22%)	9 (25%)	3 (15%)		1	11 (20%)	8 (22%)	3 (15%)	
< 1	12 (22%)	7 (19%)	5 (25%)		< 1	13 (23%)	8 (22%)	5 (25%)	
**Place of diagnosis**					**Place of ART service**				
MOH	26 (47%)	16 (44%)	10 (50%)	0.675	MOH^a^	28 (50%)	17 (47%)	11 (55%)	**0.043**
NGO/CBO	22 (39%)	16 (44%)	6 (30%)		CHA^b^Z	2 (4%)	1 (3%)	1 (5%)	
Outreach	4 (7%)	2 (6%)	2 (10%)		NGO	21 (38%)	17 (47%)	4 (20%)	
Private	3 (5%)	2 (6%)	1 (5%)		Private	5 (9%)	1 (3%)	4 (20%)	
Born with HIV	1 (2%)	0	1 (5%)						
**GAD-7**					**CESD-10**				
Low	29 (52%)	18 (50%)	11 (55%)	0.110	Low (≤ 10)	31 (55%)	22 (61%)	9 (45%)	0.422
Moderate	17 (30%)	14 (39%)	3 (15%)		Moderate (11–5)	20 (36%)	12 (33%)	8 (40%)	
High	6 (11%)	3 (8%)	3 (15%)		High (16–25)	4 (7%)	2 (6%)	2 (10%)	
Very high	3 (5%)	1 (3%)	2 (10%)		Missing	1 (2%)	0 (0%)	1 (5%)	
Missing	1 (2%)	0 (0%)	1 (5%)						

^a^ MOH: Ministry of Health

^b^ CHAZ: Churches Health Association of Zambia

### Effects on mental health

The range of stigma-related effects on the participants - included a negative influence on mental health as well as other emotional, social, or physical harms, and an increase in anxiety about recurrent experiences in the future.

### Symptoms of depression

From the responses, over half of the participants (55%) had a score < 10, indicating that they had low to no symptoms of depression while 36% had moderate to significant symptoms of depression and 7% had major depression. This proportion was higher for the MSM participants (61%) compared to the TGW participants (45%). It was also observed that a greater proportion of TGW participants had moderate to significant symptoms of depression (40%) or major depression (10%) compared to the MSM participants, at 33% and 6% respectively (*X*^2^=0.65; *p* = 0.42). These results were statistically non-significant, possibly due to small sample sizes in the study.

### Thoughts and experiences of suicide

**Fig. 2:**
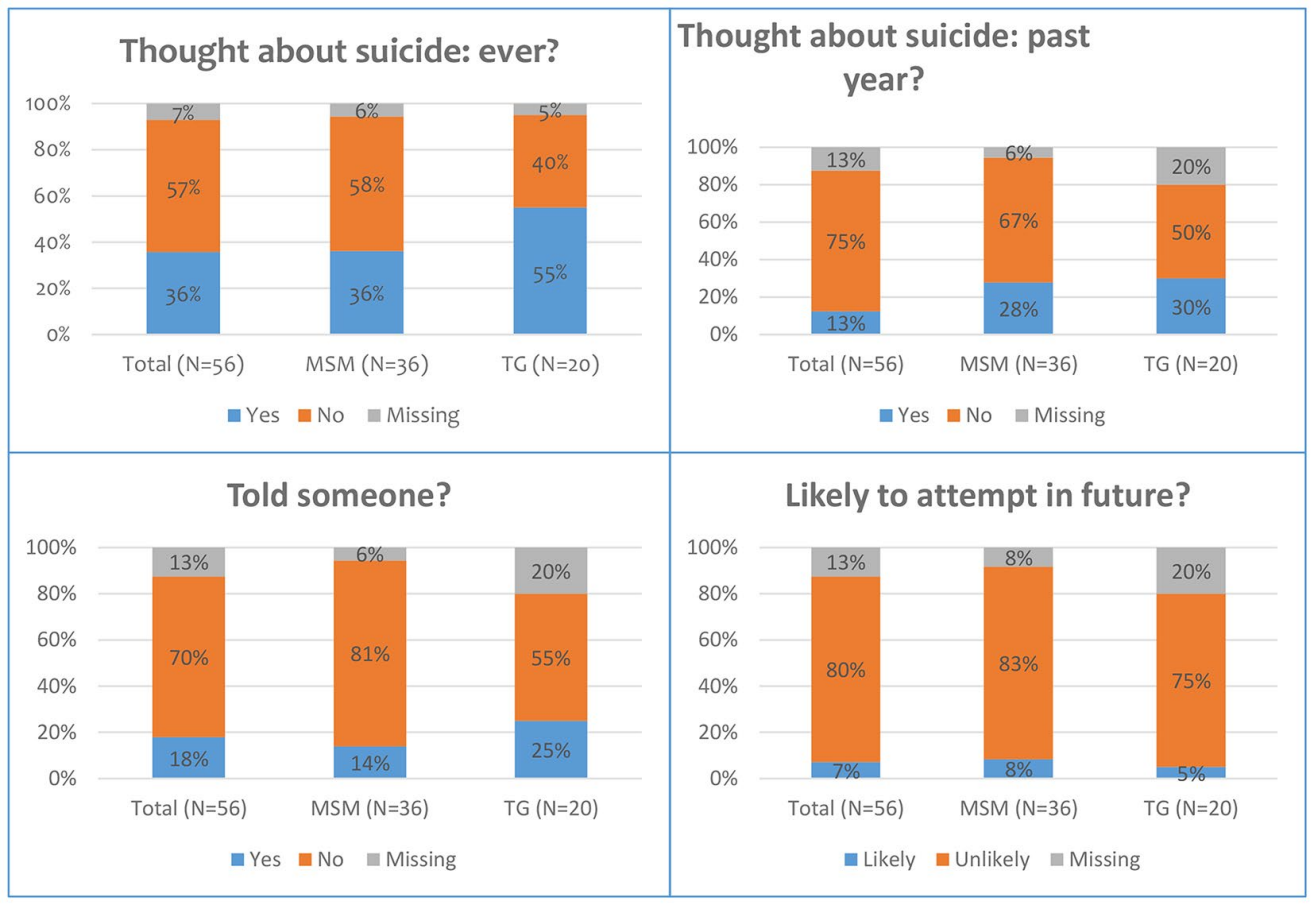
SBQ-R results

The questionnaire further inquired about thoughts and experiences of suicide using the SBQ-R tool. The results are shown below (Fig. 2):

The study found that 57% of all participants had never contemplated suicide. Of the remainder, 36% had contemplated suicide at least once, and 13% had done so in the previous year. TGW participants (55%) had contemplated suicide more than their MSM peers (36%) (*X*2 = 1.87; *p* = 0.17). Few had disclosed such thoughts to others (18% for all participants, 25% for TGW). Finally, four (7%) of the young participants (three MSM and one TGW) indicated that it was likely they may still attempt suicide at some point in the future. These states of mental health and what influenced them were also explored in detail in the qualitative results. In addition, the study found that 30% of the all participants had moderate signs of anxiety, 11% had high signs of anxiety while 4% had very high signs of anxiety. Of these participants, 11% of MSM and 25% of TGW reported high or very high GAD-7 scores (*p* = 0.110). It should the noted that the above trends were *statistically non-significant*, possibly due to small sample sizes in the study.

### Qualitative results

This section presents qualitative findings of countries about the forms, manifestations and effects of intersectional stigma among young, HIV + MSM and TGW in Zambia. The first part presents results on intersectional stigmas - origins, experiences, and fears. The emergent themes of the nature and extent of these experiences include (1) HIV, sexual orientation and gender identity disclosure; (2) Dual identity; (3) Challenges of finding and maintaining sexual partners; (4) Coping and resilience.

### The nature and extent of these experiences

Anxieties about, or experiences of stigma influenced disclosures and sometimes lead to complex arrangements for managing who could know what about them, whether about their sexual orientation, gender identity or health status. There were a number of differences between participants in terms of what they chose to disclose, why and to whom. There was as similar range of differences in terms of the reactions and consequences of such disclosures. For many participants, disclosing information about SOGI had much higher risks than disclosure about health status; perhaps an expected result given the Zambian context where HIV has a greater degree of public visibility and acceptance. This was not the case for all participants, however.

### HIV, sexual orientation and gender identity disclosure

While a small number of participants had disclosed their sexual orientation or gender identity to almost no one beyond sexual or romantic partners, others had shared this information and had expressed a degree of need to do so. As one participant stated when responding to the question why he had shared his sexual orientation with (highly) selective friends and family members:

*Because I was being suffocated with my secret! [ZMA-LSK-MB-MSM5]*.

For this participant, since he felt that his identity would eventually reveal itself, it was important for him to take the first step and to share it with others he trusted, including members of his family. For both the above participants, actively disclosing their sexuality to family and friends was an important, affirming step, despite the risks.

*Because I love myself the way I am, that’s why I told them…. It wasn’t easy. They were suspecting, so I thought through it and just ended up telling them. But it took some time, but in the end they accepted. [ ZAM-LSK-MB-MSM1]*.

With regard to disclosure of HIV status, while the reasons and circumstances for sharing or not sharing information were different, they were only slightly less difficult for many participants and still carried real or feared risks of different types of harm linked to stigma. The reasons given by participants to disclose their HIV status were often pragmatic. Some had shared information about their HIV status with others so that they could be better supported, including being reminded to take medication, or being helped with collecting it from health facilities. Others had shared their HIV status to be assured of emotional, financial, and physical support from family members, friends, or partners. For some, sharing information about their health status was a practical move, something to be done for good reason:

*Because they [family members] need to know. You may find that they discover and get surprised that I am on treatment. They need to know my status. They need to know everything about me. [ZAM-LSK-MB-MSM1]*.

Similar to information about their SOGI, some participants were more selective and careful with whom they told and why. The reasons for these considerations were, for the most part, the avoidance of stigma, as well as availability of a supportive and accommodative family environment as this TWG participant explained:

*It’s my family because they are aware of this. And they give me courage to push and be strong. Maybe it was not difficult for them to accept because one of my siblings was born with HIV. So they accept me and force me to drink.[ZAM-LSK-NN-TGW-02]*.

Meanwhile, once information began to circulate in this wider social context, whether initiated by the participants themselves, or happenings despite their efforts to conceal or keep these things hidden, the influence of stigma and the fear surrounding it intensified;

*Ah challenges. The most painful thing is you know you are in a group people are just talking about HIV and so on. You know you feel that guilt to say maybe these people know about me or maybe someone told them -----, so you just feel that and it will be paining inside you, you feel it just in you.[ZAM-CHP-NN-TGW-03]*.

This situation sometimes lead to complex arrangements for managing who could know what about them, whether about their SOGI or health status. As a result of these concerns, in some cases, people who received information about their health status were different from who received information about sexual orientation or gender identity. As shown below, the respondent thought that friends would not accept his HIV status, family would not accept his sexual orientation, so the two identities and groups must remain distinct at all times. This type selective disclosure was classified as “*two persons in one.”*

*It’s very easy, actually. Okay, I can’t say it’s easy. Those that know about my sexual orientation are friends from Lusaka and basically I am in Lusaka for school and when I am in school they know about my sexual orientation but not my HIV status. When I go back home, they know about my HIV status, but not my sexual orientation. So I, like, I am two persons in one. --MSM, 22 years, Lusaka, Zambia. [ZAM-LSK-CW-MSM6]*.

Participants explained that they struggled on a daily basis to carefully assess individuals before disclosing either their HIV status or SOGI or both. There was a skill that was acquired to read *“the way someone looks”* and their level of *“maturity”* that makes it safe or not to share information. *“Manners and behaviour”* determined who could keep the *“secret”* of an individual’s identity and their HIV status:

*I only see someone who is close to me and someone who is matured enough, or someone who can really understand, that is when I can disclose to say no… Not someone who is immature, they start telling everybody, no this guy is on medication, this guy is on this, this guy, eh. [ZAM-CHP-MB-MSM9]*.

The burden or cost of hiding both HIV stigma and SOGI could become unbearable. The need to compromise, to hide, had a weight that accumulated to the extent that it pierced and deflated self-confidence and self-acceptance. Constant denial in social setting can lead to a more profound internal denial. The trajectory from external to internalised stigma is clear in the example below.

*So, it affects me because you cannot, like, live the whole lot of your life hiding, yes? So, it does affect me a lot, yes….It affects me psychologically, sometimes I feel like denying myself, It affects me physically, mentally. -- [ZAM-CHP-MB-MSM1]*.

Intersectional stigma was also evident in norms around sexuality and HIV status and ART uptake. HIV disclosure especially to family members and close friends was a challenge as these people had never seen them with girlfriends. A participant shared his account of people would potentially attribute SOGI in this case being gay as the reason for being HIV positive. For them, it was important to hide including not telling the truth when asked about sexual orientation in order to maintain social support and reduce stress.

*It comes to my sexual orientation, it’s kind of difficult for me to tell them that I am attracted to men because what if they send me away from home? That will be difficult. I might get stressed and even stop maybe taking my drugs and then my family members will conclude that it’s because of this (gay) maybe which led me to have HIV. [ZAM-LSK-CW-MSM9]*.

Participants reported that they preferred to keep their HIV status to themselves as disclosing it would indirectly lead to disclosing one’s sexual orientation, and possibly lose social support. The difficult to openly disclose SOGI made some respondents fail to also disclose their HIV status as in this example.

*I wouldn’t even dare to tell my uncle. He is too tough. He might just ask me, ‘I have never seen you with a girl, so where did you get this from? [ZAM-LSK-NN- TGW 3]*.

For the young participants, intersectional stigma was indeed a potent force in their contexts, one that was experienced with intricate variations in its forms, manifestations, intensities and effects across many respondents. Handling the double burden of SOGI and HIV stigmatization from society was in general mentally challenging as they always feared to be laughed at and blamed by society. Due to fear of experiencing this double stigma, some participants reported that they preferred not to disclose that the they were gay and also living with HIV- a situation on one respondent classified as a *“a private lie”*.

*It’s not easy am telling you. I don’t just come out in open and tell people that am HIV positive. I don’t. I do hide myself. Reason being scared of the society they will start laughing at me, pointing fingers at me…. So its like****am living like a private lie****where I have to be hiding for who I am (MSM). It’s like I just can’t come out and tell people that am HIV positive, am gay.*— *[ZAM-SOL-WC-MSM-06]*.

### Dual identity

For those who reported experiencing stigma, traces of intersectional stigma emerged in how participants described themselves, as sexual minorities and individuals living with HIV. Some described how the awareness of being HIV positive at the time when they still struggling to accept their SOGI complicated the self-acceptance process as explained by this young man.

*So at first before I was even diagnosed having HIV I, you know, I had negative thoughts about my sexual orientation …I thought I wasn’t normal …… and then I looked away when I discovered I had HIV, I had two burdens I had my sexual orientation and I had the HIV status so I thought why have two problems …. I had anxiety I because I was thinking a lot I didn’t know what will what will become of my life*. *[ZAM- LSK- MB- MSM- 03]*.

Another MSM described the burden or struggle of living and accepting both the HIV and SOGI as living a ‘cursed’ life.

*It’s really hard. At times you just feel maybe it’s a curse and you having in this world and here is you trying to accept your sexual orientation and you are HIV positive so it’s hard*. *[ZAM-LSK-WC-MSM-02]*.

Some young people narrated that the HIV status had brought an additional challenge on top of the law that criminalizes SOGI. The burden of managing or navigating issues related to both the criminalization of SOGI and their HIV status was explained by one MSM who hoped that things can change for better in the future.

*It even becomes worse now, because in Zambia they have not legalized gay rights, and on top of that, you have HIV. So, it’s a burden on me unless maybe in the future, if God allows, that they legalize gay rights, then at least I will have one less burden! --MSM, 23 years, Lusaka. [ZAM-LSK-WC-MSM1]*.

A few explained that having to hide both identities was emotionally stressful as the they could not trust anyone with information about any of their identities

*Especially my emotions, I never wanted to trust anyone. I was stressed, my emotions were bad -…Even my physical experience, I started getting fat. I get fat whenever I’m stressed*. *[ZAM-LSK-NN-TGW-02]*.

The degree to which they accepted these things about themselves, the influence of stigma became more prominent as they expressed themselves in their social environments, beginning with who they told about themselves, what they disclosed and why. Anxieties about, or experiences of double stigma influenced these disclosures with some of them stating that both HIV and SOGI were the same, and preferred to hide both.

*There is nothing simple, they are both the same. Since you need to hide for both. You just can’t randomly tell someone that you are on medication and you also just go and tell someone who is not gay that you are gay. So, these things are the same*. *[ZAM-SOl-CW-MSM-07]*.

Although participants gave a range of descriptions with regard to these identities, most were anchored in a strong sense of understanding and acceptance that SOGI were a fundamental and enduring part of who they were as persons. This is how one young participant described himself:

*I was born like this and there is a purpose as to why I am like this. So, whatever people might say about me is not what or who I am. I am who I am today. [ZAM-LSK-CW-MSM5]*.

For another respondent, his sexual orientation had a *“purpose”* that is given at *“birth”* and this anchors his confidence and assurance: *“I am who I am today”* and not anything else. Another participant had a similar level of self-assurance:

*I am gay and I love who I am, because it’s something I didn’t just come up with, but it’s something that I feel is in me and I was born with it…So, I feel okay with it myself.-- [ZAM-CHP-MB-MSM8]*.

A TGW had a similar level of confidence, as in this example:

*Being a TGW, it is not something that you just wake up today and just say, ‘I am a TGW.’ No, it is about the way you feel yourself. The way I feel myself it is important. I feel like a woman; I see the woman in me. So, it is very important to me.-- [ZAM-LSK-NN- TGW 3]*.

Similarly, some respondents did not struggle living with HIV. The fact that there are family, friends and others also living with HIV brings a sense of solidarity and hope and greatly facilitates accepting and integrating one’s health status into one’s self identity. Those that had initially struggled to accept their HIV status also described how social support from health workers, friends and family members greatly facilitated acceptance. Health workers provided support when young people visited the health facilities. Health workers also played a key role to encourage them to start taking HIV medication as in this example:

*Because the person I found [at the health facility] counselled me about how people who are living with HIV might also survive, explaining how many (medication) to take…So I said, let me try, just try. [ZAM-SOL-WC-MSM8]*.

### Challenges of finding and maintaining sexual partners

Romantic and sexual relationships for all young people are important, regardless of SOGI or health status. They propel personal development and help young people to know themselves. For the study participants, finding and keeping partners was critical to coping and resilience. Being rejected was challenging and contributed to mental health risks. What was surprising in the data was how much of the stigma and rejection was driven by peers who should otherwise be less prejudiced or fearful. Participants reported that it was hard to balance the tension regarding finding romantic and sexual partners given the complex legal context, and HIV risk of stigma from peers.

*The challenge… is that it is difficult to balance the two because while you are thinking about HIV you also have to think of having sex with your fellow men.[ZAM-CHP-MB-MSM-02]*.

They reported that if not well managed disclosing ones HIV while trying to get into a sexual relationship could damage social reputations, limit sexual or romantic attractiveness in an environment with limited sexual options, and lower self-worth amongst the participants as explained by one TWG who was into sex work.

*It is an embarrassment for every person to know that you are on drugs (ART) ….because my business will be affected as people will run away or avoid being with me. So my business can be affected in so many ways like men who want to sleep with me they can avoid me once they know that I am HIV positive. [ZAM-LSK-TC-TGW-04]*.

This risk of intra-community stigma and its consequences appeared both more potent from sexual minority peers. Some participants struggled accepting themselves and integrating their HIV status with the tension regarding finding romantic and sexual partners given the complex legal context, which made some participants to pretend that they were HIV negative whenever they were with their partners, as this young man explained.

*Being a [gay] young man living with HIV is very, very hard. There come people that would really want to start a stable and nice relationship with you. And then there is just something there in your heart that will tell, ‘Okay, should I tell this person I am HIV or I shouldn’t? Should I just play along?’ And then it’s very hard. That person is also a human being….I hide my status because I never want to lose the people that are dear to me. -- [ZAM-LSK-MB-MSM5]*.

Some young HIV-positive MSM and TGW narrated that they always worried that people might know that they were on ART. One person experienced involuntary disclosure (outing) by her boyfriend:

*I felt bad, he spilled the secret because he drinks and if he was negative, I would have ended our relationship from there but I understand him, that’s how he is. [ZAM-LSK-NN-TGW-02]*.

Respondents reported that they feared that people within the community might make fun of or stigmatize them once they knew that they were HIV positive which could result into rejection within the relationships.

*I disclosed it to someone I had met, who I thought we would be together forever. I decided to let them know that I insist on using condoms because I am on medication. The person didn’t take it well, he said “Ah okay, but we just have to part ways. [ZAM-LSK-CW-MSM-10].*

Rejection from the current partner or potential partner was painful and could trigger suicidal thoughts as explained below.

*My partner wasn’t comfortable with me living with HIV, he was like I can’t date you this and that but I was like no, it wasn’t my fault but never the less it happened. So, it’s up to you to accept me or leave me because I don’t mind, this is the way I am and later on he started having like this mind of thought to say oh I feel like killing myself*. *[ZAM-LSK-CW-MSM-05]*.

### Coping strategies and resilience

Many respondents reported that they had developed different positive ways of coping with the effects of stigma. These positive coping strategies helped them to cope with many mental health challenges that they experienced. These positive experiences included exercise (sports, walking, and swimming), meditation and prayer, reading, or just keeping busy, as in these examples:

*I work out, I go to the gym, I do a bit of meditation, I read certain books that usually sharpen my mental faculties. [ZAM-CHP-MB-MSM8]*.

*I worship my Lord, and I know that everything is possible with Him by my side. [ZAM-CHP-NN- TGW 2]*.

A TGW explained that she managed the mental health challenges through undertaking many duties at home. By keeping busy, she was able to keep her mind focused and refreshed as explained below.

*I keep my mind off things that are making me feel bad and I do different kinds of chores around the house, to remove the pressure I have. [ZAM-LSK-NN- TGW 4]*.

Focussing on positive or purposeful activities relieved stress or lifted the burden of poor mental health for these young people. Staying sexually activity was also important as explained below:

*So, when I have sex, it helps me to think better. [ZAM-CHP-MB-MSM2]*.

Finally, some participants had very positive and empowering ways of recovering from stress or poor mental health. These attitudes and practices put them in a position of becoming resilient and being able to resist the negative aspects of their experiences as sexual minorities and as PLHIV. This young participant found such resilience after recovering from a violent attack:

*It [the attack] affected in a way that they attacked me and I didn’t like it. But it also strengthened me such that when someone passes a comment, I can stand and say, ‘So!?’ I can just tell them what they want to hear. I would say, okay this is me, if you won’t accept me, bypass me. [ZAM-LSK-WC-MSM10]*.

Another young person found a way to become more self-reliant and handle his own issues. He explained that he did not like to involve other people in his problems as they might think that he is not resilient enough to address the problems.

*I handle my issues on my own. I do not like to involve too many people because they can look down on me that, every day, I have issue, like I’m always having the same issues, every day. So, you will find that I handle my issues on my own in whatever situation I am in. Unless when the issue is too big that is when I can go to a counsellor. [ZAM-LSK-WC-MSM4]*.

A few narrated that they were able to cope by taking to friends who work with NGOs that provide services to young key populations. Such discussions helped the young key populations to manage the challenges associated with being HIV and MSM as explained below.

*First of all, it makes me feel terrible about myself and it also made me feel I amount to nothing because am now a person living with HIV and I have this sexual orientation going on so it’s really hard but then with the help of these Non-governmental organizations I have friends that I can talk to that really understand yah. [ZAM-LSK-WC-MSM-02]*.

This individual found a way to be immune to disturbances. A TGW participant explained that she preferred not to think too much about what people say about her as doing so might stress her much more.

*I just sit back and relax, and not think too much about people, because one day they might get sick as well. Because we are all sick in the world, nobody is perfect. So, I just sit back and relax, and cool myself down, and say to myself that everything will just be okay, yah.[ZAM-LSK-TC- TGW 2]*.

## Discussion

This study aimed at exploring how intersectional stigma is constructed among young, HIV-positive MSM and TGW in Zambia and how it affects mental health outcomes. This is one of the rare studies done in Zambia on MSM and TGW on intersectional stigma, depression and suicidal thoughts. The study showed that a convergence of multiple stigmatized identities or experiences among MSM and TGW affected their mental health. Most of the participants experienced dual stigma, that is SOGI stigma and HIV stigma. More TGW participants had moderate to significant symptoms of depression (40%) or major depression (10%) compared to the MSM participants, at 33% and 6% respectively. Similarly, more TGW participants (55%) had contemplated suicide than MSM (36%). These findings align with a literature review on intersectional stigma which demonstrated that intersecting forms of stigma can impact mental and physical health, as well as related health behaviors [45]. In this context, Logie et al. [46] have mapped the relationships between intersectional stigmas, depression, and adverse HIV outcomes among HIV-positive women in Canada which they assert can be synergistic and compounding.

Stigma coupled with the existence of the law that criminalize same sex relationships, made some MSMs and TGW not only to hide their HIV status and ART but also their SOGI. Thus, in the context of this study, intersectional stigma was shaped by prevalent views and perceptions that a given identity (SOGI) and respective HIV status was deviation from accepted social norms. Each identity worsened levels of stress, and vulnerability levels among MSM and TGW. These findings align with a recent scoping review of HIV-related intersectional stigma among sexual and gender minorities in sub-Saharan Africa [47]. We thus agree on the need for increased recognition of how an individual’s membership in multiple stigmatized groups including HIV-related stigma may not only be a *manifestation of fears related to the health condition itself, but also negative attitudes regarding behaviors and identities originally associated with HIV transmission including sexual orientation* [48].

The double burden of hiding SOGI and HIV status from relatives, friends, partners and community members triggered loneliness, more stress, loss of self-esteem, anxiety, depression and suicidal thoughts among MSM and TWG. We note that if not well managed, these mental health challenges could undermine HIV self-management behaviour among MSM and TGW which could affect both the physical and mental wellbeing. This finding corresponds with previous research that has documented an association between HIV-related stigma experienced by young MSM in Chicago and other psychosocial factors related to HIV (i.e., psychological distress, lower self-esteem, loneliness) [49].

The findings suggest the existence of a social structure which constrains young, HIV-positive MSM and TGW’s ability to freely live out their HIV status, take and adhere to ART, as well as disclose their HIV status and SOGI. Such a limiting social structure could affect their health status not only by reducing their ability to adhere to treatment but also constrain safe sexual practices. Examples of unsafe practices included some participants in this study reporting engaging in unprotected sex and not disclosing their HIV status to their partners for fear of losing them. We thus conclude that this discriminatory social structure is problematic as it limits MSM and TGW’s agency which is ‘the capacity to transpose and extend personal schemas to new contexts [50], including disclosing their HIV status and identity to new sexual partners, friends, health workers and in some cases family members.

Further, an excluding structure could have negative implications on MSM and TGWs’ ability to confidently define, understand and accept their HIV positive identity, and live positively given that significant others, such as partners, friends, health workers are the key drivers of stigmatization. It has been argued that ‘feedback about one’s position can provide a sense of security or sense of threat to self’ [51]. Thus, feedback in the form of stigma and mistrust, can over time make the MSM and TGW living with HIV to view themselves as lesser members of the family, school and society. Hence, it is crucial for interventions targeting intersectional stigma to emphasize the significance of thoroughly examining the impact that stigmatizing language, behaviors, and attitudes can have on individuals, potentially leading to the categorization of individuals into “us” and “others” [52]. In this study, we note that this classification could have strongly led to a limited sense of belonging to their families by some MSM and TGW and possible suicidal thoughts or a wish of dying from AIDS by stopping ART.

These excluding social structures could also make the young participants not chose to access social support, including from other MSM or TGW peers. As a way of responding to these negative mental health outcomes, MSM and TGW adopted different forms of positive or wellbeing enabling coping strategies. The positive coping strategies included exercising, listening to music, singing, chatting and praying. We also note that others reported adopting negative (dysfunctional) coping behaviours, a finding which is similar to another study on mental coping behaviours among young MSM in Zambia [10].

### Strengths and limitations of the study

The adoption of the mixed study design enhanced the strength of the study. Triangulating data collection methods helped in developing a comprehensive account of experiences of intersectional stigma and mental health challenges among young MSM and TGW living with HIV. Credibility of the findings was enhanced through thoroughly documenting the research process including recruitment, as well as data collection, entry, transcribing and analysis processes. In addition, sharing the results with stakeholders including young TGW and MSM living with HIV helped in clarifying and validating the findings. Our findings cannot be generalized to whole country as the participants in our study were only drawn from three provinces. Further the sample size was small as it consisted of 56 participants. Despite these limitations, the study provides useful information to enhance programming for young TGW and MSM living with HIV as there is dearth of such studies in Zambia and arguably across much of Africa that address the many challenges that key populations face on a day-to-day.

## Conclusion

Most young HIV-positive MSM and TGW experienced intersecting forms of stigma at household, community and health system levels. The intersectional stigma was rooted within different salient historical, cultural, and socioeconomic contexts. Fear of disclosure of both SOGI and HIV status from relatives, friends, partners and community members triggered poor mental health outcomes which included anxiety, depression and suicidal thoughts. Socio-cultural and religious attitudes regarding SOGI and criminalization of sexual or gender diversity in Zambia also contributed this intersectional stigma. In trying to respond to these mental health challenges, young, HIV-positive MSM and TGW adopted several positive coping strategies. To address intersectional stigma and related mental health challenges, we recommend strengthening implementation of laws and policies that provide a favourable environment for MSM and TGW living with HIV, including stigma reduction policies that ameliorate the negative impacts of multiple intersecting stigmas. There is also a need to meaningfully engage MSM and TGW who are at the centre of experiencing these intersectional stigmas, as well as political and community leaders in the development of interventions aimed at addressing social, cultural and legal practices that lead to stigmatisation. We further recommend mixed methods implementation research on community based interventions for reducing mental health challenges among young, HIV positive MSM and TGW in an African setting. These interventions should focus more on trauma-informed and identity-supportive care for young people with HIV.

## Data Availability

The datasets during and/or analysed during the current study are available from the corresponding author on reasonable request.
